# Effects of walking speeds on lower extremity kinematic synergy in toe vertical position control: An experimental study

**DOI:** 10.1097/MD.0000000000038024

**Published:** 2024-05-03

**Authors:** Xuan Liu, Jitong Liang, Ye Liu

**Affiliations:** a Beijing Sport University, Beijing, China.

**Keywords:** joints, kinematics, stability, synergy, walking

## Abstract

**Background:**

This study aimed to investigate whether lower limb joints mutually compensate for each other, resulting in motor synergy that suppresses toe vertical position fluctuation, and whether walking speeds affect lower limb synergy.

**Methods:**

Seventeen male university students walked at slow (0.85 ± 0.04 m/s), medium (1.43 ± 0.05 m/s) and fast (1.99 ± 0.06 m/s) speeds on a 15-m walkway while lower limb kinematic data were collected. Uncontrolled manifold analysis was used to quantify the strength of synergy. Two-way (speed × phase) repeated-measures analysis of variance was used to analyze all dependent variables.

**Results:**

A significant speed-by-phase interaction was observed in the synergy index (SI) (*P* *<* .001). At slow walking speeds, subjects had greater SI during mid-swing (*P* *<* .001), while at fast walking speeds, they had greater SI during early-swing (*P* *<* .001). During the entire swing phase, fast walking exhibited lower SI values than medium (*P* *=* .005) and slow walking (*P* *=* .027).

**Conclusion:**

Kinematic synergy plays a crucial role in controlling toe vertical position during the swing phase, and fast walking exhibits less synergy than medium and slow walking. These findings contribute to a better understanding of the role of kinematic synergy in gait stability and have implications for the development of interventions aimed at improving gait stability and reducing the risk of falls.

## 1. Introduction

Walking is a common and necessary activity in most people daily lives, and gait patterns are characterized by subtle variations in gait spatiotemporal parameters and leg swing trajectories.^[1–3]^ These variations can arise from motor abundance, which allows for multiple variations in joint and muscle coordination to achieve a given task performance.^[4]^ For walking, coordination among multiple joints is required to achieve precise end-point motor control to maintain efficiency and smooth walking.^[5]^

The extent to which motor abundance is exploited to accomplish a motor task can be evaluated using the uncontrolled manifold (UCM) method. It can provide information on how variations in all degrees of freedom (DOFs) (termed elemental variables) contribute to performance and co-vary to produce consistent output in a performance variable, resulting in motor synergy.^[6–8]^ The UCM method quantifies the variability of elemental variables across repeated trials and splits them into 2 components: variance within the UCM (V_UCM_), and variance orthogonal to the UCM (V_ORT_). V_UCM_ represents “good variability,” which can freely vary to solve the movement task flexibly without leading to increased variability in task performance or changes in the values of the task variable. For example, several combinations of shoulder, elbow, and wrist angles lead to the same hand position. Thus, V_UCM_ contributes to the stability while affording flexibility. In contrast, V_ORT_ represents “bad variability,” where some combinations of elemental variables can interfere with the stability of the performance variable. The difference between the 2 variance indices, V_UCM_ and V_ORT_, was used as the synergy index (SI). A positive SI value indicates that V_UCM_ is larger than V_ORT_, reflecting the strength of kinematic synergy. This means that many elemental variables use motor abundance to stabilize a specific performance variable.^[6]^ In other words, if 1 DOF introduces an error into the system, the other DOFs will respond so that the performance is not compromised.

During human walking, the foot serves as the end-effector of a system with multiple DOFs. Previous UCM analyses of foot swing trajectories have mainly focused on the mediolateral direction,^[9–11]^ as they are sensitive to perturbations in the frontal plane.^[12–14]^ However, a reduction in foot vertical position leads to tripping while walking, which is a major cause of falls.^[15]^ In healthy individuals, the foot vertical position is mainly achieved by limb shortening, which is affected by hip flexion, knee flexion, and ankle dorsiflexion.^[16]^ Therefore, it is important to investigate whether there is a synergistic effect of the lower limbs in stabilizing the vertical direction of the foot trajectory.

Walking speed is a critical factor that affects various aspects of gait, including joint kinematics, ground reaction forces, and muscle activity.^[17,18]^ Despite the importance of this factor, the effects of walking speed on the motor control strategy of the central nervous system (CNS), which coordinates multiple variables to stabilize important performance variables, have not yet been fully elucidated. However, some studies reported inconsistent results. For example, Monaco et al^[19]^ found that the organization of lower limb joint variance underlying the stabilization of the center of mass position remains almost unaltered across speeds. In contrast, Kao et al^[20]^ found that the kinematic synergy in footpath stabilization is stronger at slower speeds than at higher speeds. Tokuda et al^[21]^ identified differences in the kinematic synergy in the center of mass displacement stabilization at different speeds. Therefore, further investigation is needed to determine whether walking speed affects lower-limb synergy, which in turn affects the foot swing trajectory.

The purpose of this study was to investigate whether the fluctuations in lower limb joint trajectories compensate for each other to stabilize the toe vertical position during the swing phase, using UCM analysis. Furthermore, we aimed to explore whether there were any speed-related differences in these kinematic synergies. In this study, the walking speed was set according to the Froude (Fr) number. Fr is a dimensionless parameter, which was initially designed in order to normalize the same movement in subjects of different sizes.^[22,23]^ In addition, to avoid the influence of the treadmill on gait performance, this study was conducted on a flat ground for the walking experiment. Therefore, the control of walking speed during the experiment needs to be as precise as it is. Some studies provide evidence that the mediolateral trajectory of the foot is stabilized by kinematic synergy and has been reported in healthy, aged and disabled subjects.^[9–11,20]^ While Yamagata et al^[11]^ demonstrated that the synergy stabilizing the swing foot in the vertical direction is affected by fall history, they did not find significant differences in synergy across swing sub-phases. Moreover, Kao et al^[20]^ observed that changes in the walking speed affected the kinematic synergy differences between the swing sub-phases. Based on these findings, we hypothesized the presence of kinematic synergy in lower limb joints to stabilize the toe vertical position, and that the synergy would be influenced by different walking speeds across swing sub-phases.

## 2. Methods

### 2.1. Participants

Seventeen males (age: 23.8 ± 2.3 years; weight: 73.3 ± 7.8 kg; and height: 174.8 ± 5.2 cm) volunteered to participate in this study. The participants had no history of serious injuries or surgery in their lower extremities within the previous year. The sample size was calculated using G*power software version 3.1.9.7 (Heinrich Heine University, Dusseldorf, Germany),^[24]^ based on an effect size of 0.5, power of 0.8, alpha of 0.05 and assumptions of repeated measure analysis of variance (ANOVA) within-between interaction. The total sample size was estimated at 39 participants, with 13 participants per speed group. To allow for attrition, we recruited 17 participants per speed group.

All experimental procedures were approved by the Ethics Committee of the Sports Science Experiment of the Beijing Sport University (2021060H). Written informed consent was obtained from all the participants.

### 2.2. Procedure

Participants were asked to walk on a 15-m walkway at slow (0.85 ± 0.04 m/s), medium (1.43 ± 0.05 m/s) and fast (1.99 ± 0.06 m/s) speeds set in accordance with the Froude (Fr) number to eliminate individual differences^[22,23]^:


$$\begin{array}{l} \begin{array}{l} v=\sqrt{Fr\cdot g\cdot l}\; \end{array} \end{array}$$ (1)

where v is the walking speed in m/s, g  is the gravitational acceleration (9.8 m/s^2^) and l is the leg length from the external surface of the greater trochanter to the lateral malleolus (0.83 ± 0.04 m). Three Fr values were used: Fr_1_ = 0.09, Fr_2_ = 0.25, and Fr_3_ = 0.5.^[22]^ The Smartspeed (Fusion Sport, Australia) measurement system was used to monitor walking speeds.

Participants were asked to walk on a 15-m walkway at each of the prescribed speeds for a total of 15 trials per speed. Only 1 complete and stable gait cycle was collected per trial, and the first and last 5 m were excluded from the analysis to avoid acceleration and deceleration during walking to identify spatiotemporal gait parameters in a steady state.^[25]^ The order in which the participants walked at each speed was randomized to prevent any order effects. The order of the speeds was determined randomly by the investigator using the lottery method. Participants wore similar, lightweight, and comfortable walking shoes (Decathlon, Villeneuve-d’Ascq, France) provided by the research team throughout the experimental sessions.

As a Helen Hayes-marker set, 29 reflective-marker positions were attached to the participant body.^[26]^ The kinematics data were recorded using 8 infrared cameras (Motion Analysis Corp, USA) at a 200 Hz sample frequency.

### 2.3. Data processing

**Figure 1.**
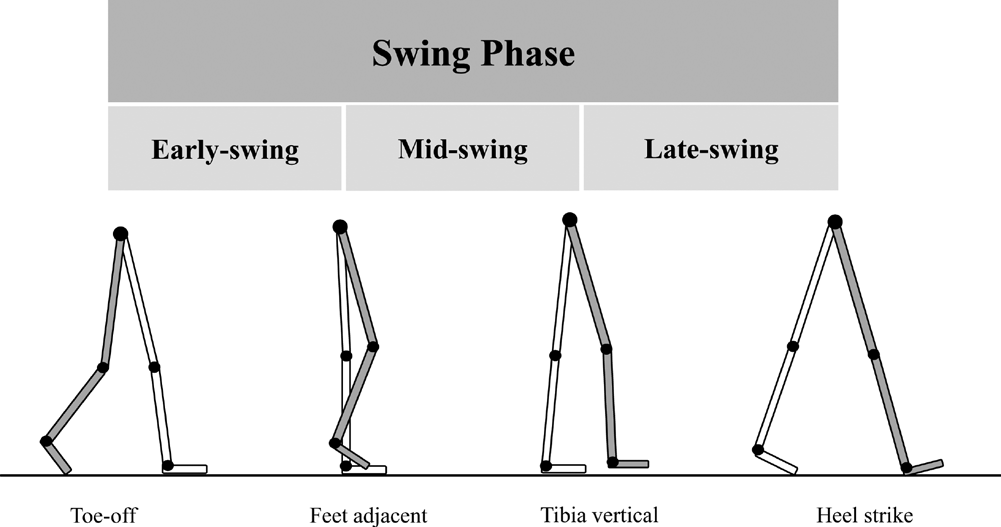
Three gait swing sub-phases. Early-swing begins with lift of the foot from the floor and ends when the swinging foot is opposite the stance foot. Mid-swing begins as the swinging limb is opposite the stance limb and ends when the swinging tibia is vertical. Late-swing begins with a vertical tibia and ends when the foot strikes the floor.

Marker trajectories were identified using Cortex 2.6 motion capture software (Motion Analysis Corp, USA) and smoothed using a low-pass, fourth-order, and zero-lag Butterworth filter with a 13.3 Hz cutoff. Only the kinematic patterns in the sagittal plane were examined in this study. The main gait events, heel-strike and toe-off, were identified from the kinematics of foot markers to classify the swing phase. It was difficult to divide the swing data individually using kinematic data; therefore, we divided the swing data equally into 0% to 33% (early-swing), 34% to 67% (mid-swing), and 68% to 100% (late-swing) periods (Fig. 1).^[27]^ Each swing cycle time series was time normalized to 101 data points which were then used to implement the UCM.

### 2.4. Uncontrolled manifold analysis

**Figure 2.**
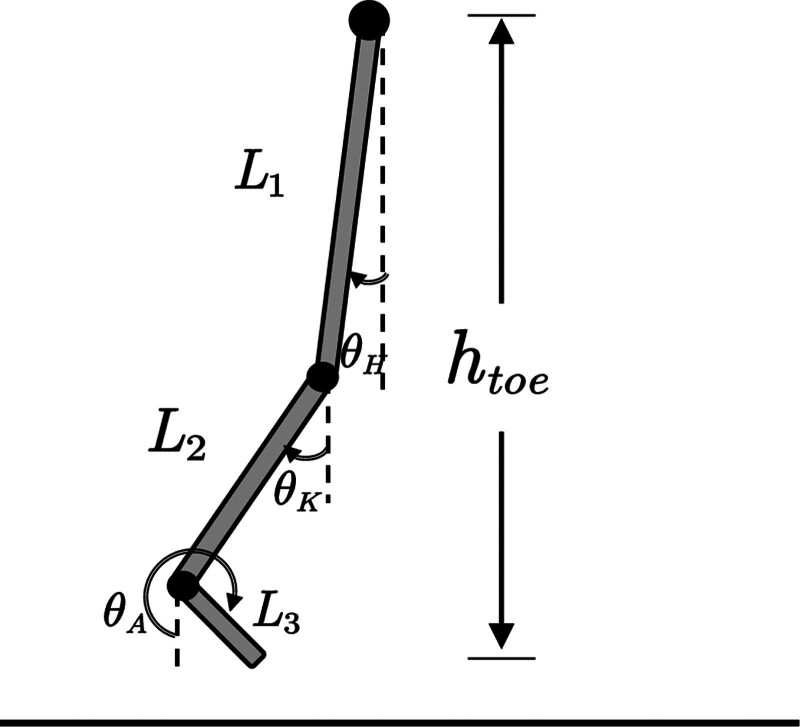
The planar model was used to estimate joint angles. $\theta_{H}$: hip angle, $\theta_{K}$: knee angle, $\theta_{A}$: ankle angle, $L_{1}$: thigh length, $L_{2}$: shank length, $L_{3}$: foot length and $h_{toe}$: vertical toe position relative to the hip position.

Based on previous literature,^[28]^ a planar model of human walking was developed, accounting for 3 body segments (Fig. 2). Briefly, the geometric model included 3 segments: swing-thigh, swing-shank, and swing-foot, with corresponding segment length of L_1–3_. $\theta_{1-3}$ are the angles between each segment relative to the vertical axis. We defined hip, knee, and ankle segment angles relative to the vertical axis as elemental variables and vertical toe position relative to the hip position as a performance variable.

The toe height is given by the joint angles:


$$\begin{array}{l} \begin{array}{l} h_{toe}=L_{1}\cos\left(\theta_{H}\left(i,t\right)\right)+L_{2}\cos\left(\theta_{K}\left(i,t\right)\right)+L_{3}\cos\left(\theta_{A}\left(i,t\right)\right)\; \end{array} \end{array}$$ (2)

where i is the joint trajectory of the i-th stride, t is the normalized time, $\;L_{1}\;$ is the thigh length, $L_{2}\;$ is the shank length, $L_{3}\;$ is the foot length, $\theta_{H}\;$ is the hip segment angle relative to the vertical axis, $\theta_{K}\;$ is the knee segment angle relative to the vertical axis, $\theta_{A}\;$ is the ankle segment angle relative to the vertical axis.

The gradient of this function was calculated by taking the average value of the elemental variables as the reference configuration, thus obtaining a vector ε  pointing to the direction of the maximum change in the 3 elemental variables. The length of the vector orthogonal to the manifold was computed by taking the square root of the dot product ε. Finally, the gradient can be normalized to a unit vector $V_{0}$:

$$\begin{array}{l} \begin{array}{l} V_{0}=\frac{1}{\left|\varepsilon \right|}\times \varepsilon \;\; \end{array} \end{array}$$ (3)

We then calculated a vector defining the deviation between the actual joint configuration and the mean joint configuration as follows:


$$\begin{array}{l} \begin{array}{l} \emptyset =\left[\theta_{H}\left(i,t\right)-\bar{\theta_{H}}\left(t\right),\theta_{K}\left(i,t\right)-\bar{\theta_{K}}\left(t\right),\theta_{A}\left(i,t\right)-\bar{\theta_{A}}\left(t\right)\right]\; \end{array} \end{array}$$ (4)

where i is the joint trajectory of the i-th stride, and t is the normalized time.

The UCM component $L_{UCM}$ and its orthogonal component $L_{ORT}$ are given by:

$$\begin{array}{l} \begin{array}{l} L_{ORT}=\left(\emptyset \cdot V_{0}\right)\times V_{0}\; \end{array} \end{array}$$ (5)

$$\begin{array}{l} \begin{array}{l} L_{UCM}=\emptyset -L_{ORT}\; \end{array} \end{array}$$ (6)

The variance of the component parallel $V_{UCM}$ and its orthogonal $V_{ORT}$ to the UCM are given by:

$$\begin{array}{l} \begin{array}{l} V_{UCM}=\frac{1}{n-d}\cdot \frac{1}{N}\sum {\left|L_{UCM}\right|}^{2}\; \end{array} \end{array}$$ (7)

$$\begin{array}{l} \begin{array}{l} V_{ORT}=\frac{1}{d}\cdot \frac{1}{N}\sum {\left|L_{ORT}\right|}^{2}\; \end{array} \end{array}$$ (8)

where n and d are the number of elemental variables and the number of performance variables, respectively.

The SI is calculated to determine the existence of joint synergy and its strength:

$$\begin{array}{l} \begin{array}{l} SI=\frac{V_{UCM}-V_{ORT}}{V_{UCM}+V_{ORT}}.\; \end{array} \end{array}$$ (9)

If the result SI > 0, then it indicates the presence of joint synergy, that is the change in the vertical position of the toe is effectively suppressed by joint synergy, whereas SI ≤ 0 indicates no synergy.^[28]^

### 2.5. Statistical analysis

Data processing and statistical analysis were performed using a custom-written MATLAB script (The MathWorks Inc., USA) and SPSS 26.0 (SPSS Inc., USA). A 2-factor repeated-measures ANOVA (walking speed and swing sub-phase effects) was used to compare the main and interactive effects. For significant interaction terms, simple effects were analyzed using Bonferroni post hoc analysis; otherwise, the main effects were considered. Statistical significance was set at *P* *<* .05, partial eta squared (*ƞ*^2^) was calculated as an effect size estimate for the ANOVA models.^[29]^ According to previous literature,^[20]^ *ƞ*^2^ values were interpreted as: 0.02 “small” effect, 0.13“medium” effect, and 0.26 “large” effect. Data are expressed as the mean ± standard deviation.

## 3. Results

### 3.1. Variance within the uncontrolled manifold

There were significant main effects for walking speed (F_(2,26)_ *=* 10.483, *ƞ*2 = .446, *P* *<* .001) and swing sub-phases (F_(3,39)_ *=* 23.213, *ƞ*2 *=* .641, *P* *<* .001) as well as a significant interaction effect for speed × phase (F_(6,78)_ *=* 10.102, *ƞ*2 *=* .437, *P* *<* .001).

**Figure 3.**
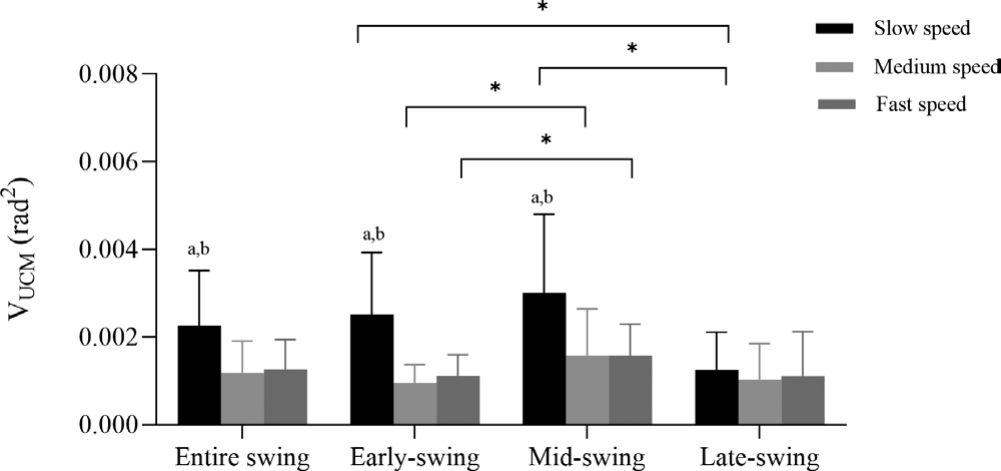
Averaged V_UCM_ across subject during the different swing sub-phases. V_UCM_: variance within the uncontrolled manifold. “*” represents statistically significant difference between 2 swing sub-phases at each speed. Different letters indicate significant differences in walking speed for each swing sub-phase, where “a” indicates a significant difference between slow and medium speed, and “b” indicates a significant difference between slow and fast speed.

Post hoc analyses indicated that subjects had significantly greater V_UCM_ in the early-swing (*P* *=* .005) and mid-swing (*P* *<* .001) than in the late-swing during slow walking. In addition, subjects had significantly greater V_UCM_ in the mid-swing than in the early-swing at medium (*P* *=* .044) and fast walking (*P* *=* .014). V_UCM_ was significantly larger during slow walking than during medium and fast walking during the entire swing phase (slow vs medium: *P* *=* .016; slow vs fast: *P* *=* .003), early-swing (slow vs medium: *P* *=* .002; slow vs fast: *P* *=* .001) and mid-swing (slow vs medium: *P* *=* .023; slow vs fast: *P* *=* .002) (Fig. 3).

### 3.2. Variance orthogonal to the uncontrolled manifold

There were significant main effects for walking speed (F_(2,24)_ *=* 4.434, *ƞ*^2^ *=* .270, *P* *=* .023) and swing sub-phases (F_(3,36)_ *=* 9.900, *ƞ*^2^ *=* .452, *P* *=* .003) as well as a significant interaction effect for speed × phase (F_(6,72)_ *=* 22.754, *ƞ*^2^ *=* .655, *P* *<* .001).

**Figure 4.**
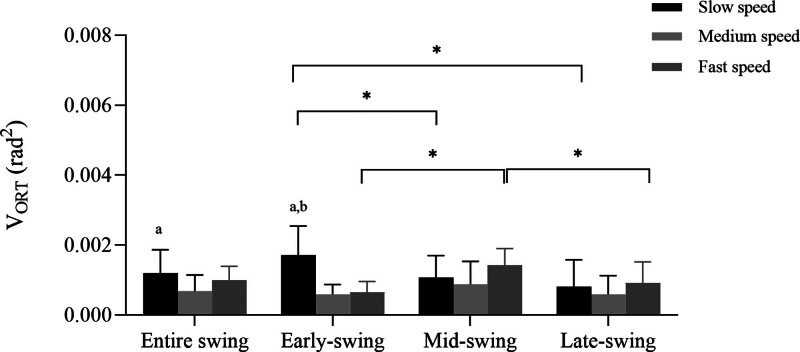
Averaged V_ORT_ across subject during the different swing sub-phases. V_ORT_: variance orthogonal to the uncontrolled manifold. “*” represents statistically significant difference between 2 swing sub-phases at each speed. Different letters indicate significant differences in walking speed for each swing sub-phase, where “a” indicates a significant difference between slow and medium speed, and “b” indicates a significant difference between slow and fast speed.

Post hoc analyses indicated that V_ORT_ was highest in the early-swing phase when walking at slow speeds (early-swing vs mid-swing: *P* *<* .001; early-swing vs late-swing: *P* *=* .002). In addition, V_ORT_ was highest in the mid-swing phase when walking at fast speeds (mid-swing vs early-swing: *P* *<* .001; mid-swing vs late-swing: *P* *=* .026). The V_ORT_ was significantly larger in slow walking than in medium (*P* *<* .001) and fast walking (*P* *<* .001) during the early-swing phase. In addition, V_ORT_ was greater during slow walking than medium walking (*P* *=* .048) during the entire swing phase (Fig. 4).

### 3.3. Synergy index

**Figure 5.**
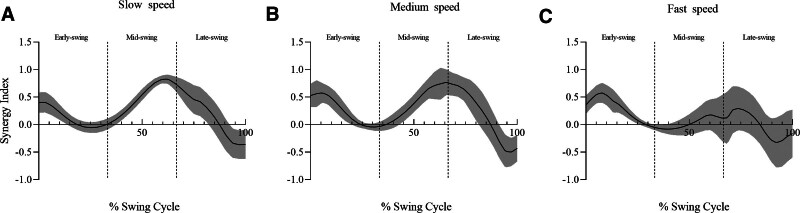
The time course of synergy index during the (A) slow speed walking, (B) medium speed walking and (C) fast speed walking, time-normalized to 100% of the swing cycle (mean in black and standard deviation as a gray area).

Throughout most of the swing phase, subjects had SI greater than zero, indicating V_UCM_ > V_ORT_ (Fig. 5). There were significant main effects for walking speed (F_(2,26)_ *=* 9.044, *ƞ*^2^ *=* .410, *P* *=* .001) and swing sub-phases (F_(3,39)_ *=* 10.405, *ƞ*^2^ *=* .445, *P* *=* .005) as well as a significant interaction effect for speed × phase (F_(6,78)_ *=* 16.429, *ƞ*^2^ *=* .558, *P* *<* .001).

**Figure 6.**
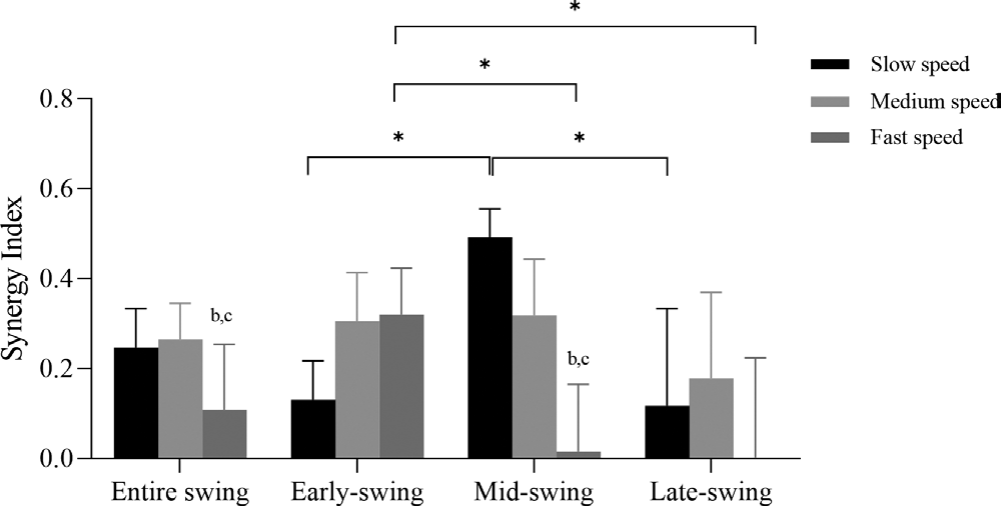
Averaged synergy index values across subject during the different swing sub-phases. “*” represents statistically significant difference between 2 swing sub-phases at each speed. Different letters indicate significant differences in walking speed for each swing sub-phase, where “b” indicates a significant difference between slow and fast speed, and “c” indicates a significant difference between medium and fast speed.

Post hoc analyses revealed that subjects had significantly greater SI during the mid-swing phase of slow walking (*P* *<* .001) and a greater SI during the early-swing phase of fast walking (*P* *<* .001), whereas SI was not significantly different across phases at medium speed. Furthermore, in the entire swing phase, fast walking presented lower SI values compared to slow walking (*P* *=* .027) and medium walking (*P* *=* .005), especially in the mid-swing phase (fast vs slow: *P* *<* .001; fast vs medium: *P* *<* .001) (Fig. 6).

## 4. Discussion

The purpose of this study was to examine the synergistic behavior of lower limb joints in stabilizing the toe vertical position during walking and how this synergy is influenced by gait speed. Our findings revealed that the toe vertical position was stabilized by a kinematic synergy during the swing phase of gait, as indicated by SI values >0. Moreover, we observed significant differences in this synergy between swing sub-phases at different walking speeds, with the lowest SI values occurring during fast walking throughout the entire swing phase especially in the mid-swing phase, suggesting a weaker synergy at faster walking speeds.

Our study findings revealed that kinematic synergy in the lower limbs stabilized the toe vertical position during the swing phase of walking, as evidenced by the significant SI values that exceeded zero. According to Remelius et al,^[30]^ the body is most vulnerable to perturbations during the swing phase; therefore, the presence of movement synergy during this phase may be a mechanism for maintaining a stable toe position during a potentially unstable time. Furthermore, the SI curve analysis showed a decrease at early-swing followed by an increase at mid-swing, with a tendency to decrease at late-swing. Owing to the toe trajectory nadir that occurs at mid-swing, termed minimum toe clearance,^[5,31,32]^ is the gait event associated with the tripping risk because the foot passes within 1.0 to 2.0 cm of the ground, and is moving at a very high velocity (4.6 m/s).^[5]^ Thus, the increase in SI values at mid-swing is likely a mechanism to prevent foot-ground contact and mitigate tripping risk. After mid-swing, feedback on the position of the foot is required to ensure sufficient foot placement at heel strike for dynamic stability. Previous studies have demonstrated that a decrease in synergy prior to postural perturbation is important for feed-forward adjustment.^[33,34]^ Therefore, the rapid decline in synergy during late-swing may be necessary for posture adjustment and preparation for appropriate foot placement at heel strike.

Our study found no significant difference in kinematic synergy between sub-phases at medium walking speed. This is consistent with the findings of Yamagata et al,^[11]^ which indicated that medium speed walking provided more balanced synergistic control of the 3 sub-phases. At slow walking speeds, kinematic synergy was strongest in mid-swing but weakest in early-swing, whereas at fast walking speeds, kinematic synergy was strongest in early-swing but weakest in mid-swing. This suggests that adjusting the walking speed leads to changes in synergy across different phases. Previous studies have shown that the increasing frequency of motor tasks leads the CNS to control the V_UCM_ and V_ORT_.^[35,36]^ In our study, slow walking had greater V_UCM_ during early- and mid-swing, and greater V_ORT_ during early-swing, indicating that walking speed can be used to modify the inherent mechanical instability of the foot position. Specifically, walking speed can increase “good variability” to stabilize foot position or decrease “bad variability” to prevent destabilization during the swing phase. In contrast to the stance phase, where V_UCM_ and V_ORT_ were not affected by walking speed,^[19]^ the swing phase appeared to be more sensitive to changes in walking speed.

In addition, our study revealed that the kinematic synergy involved in stabilizing the vertical foot position was reduced during fast walking compared to medium and slow walking. These findings were consistent with the results of Kao et al^[20]^ This adjustment of synergy was also reported in previous studies, which might be related to the instability of the movement, such as those with a history of falls or fatigue of ankle dorsiflexors had greater SI.^[11,37]^ A previous study also showed that patients with Down syndrome walked with higher V_UCM_ as well as higher positional variability. The authors interpreted that patients exploited a larger workspace and variability to compensate for poor postural control; as a result, an increase in synergy was observed.^[38]^ This suggests that fast walking is associated with stronger postural control, and thus, has lower synergistic effects.

The observed changes in kinematic synergy cannot be solely attributed to the passive dynamics of the musculoskeletal system.^[14,39–41]^ The active control of the CNS plays a significant role in mediating foot clearance through the coordination of hip and knee flexion and ankle dorsiflexion under direct corticospinal control.^[42–45]^ Age, cognitive function, attention and visual feedback all affect foot clearance.^[31,45,46]^ Furthermore, kinematic patterns are highly dependent on walking speed.^[47]^ The effect of speed on motor control of locomotion has been observed in animal studies,^[48–50]^ which have demonstrated distinct recruitment of spinal neuronal groups, depending on the speed of progression. Therefore, the observed modifications in the organization of V_UCM_ and V_ORT_ during steady walking across speeds likely reflect speed-dependent adjustments made by the CNS to manage sensorimotor related noise. Thus, it is likely that the observed changes in synergies cannot be explained by mechanics alone and point toward CNS control.

Overall, our study contributes to the understanding of the role of kinematic synergy in gait stability at different walking speeds. These findings have implications for the development of interventions aimed at improving gait stability and reducing the risk of falls, particularly in individuals with foot drop or other lower limb motor dysfunctions. Our study highlights the importance of considering kinematic synergy in the development of future interventions and rehabilitation approaches for individuals with lower limb motor dysfunctions.

### 4.1. Limitations and future work

Our study has several limitations. On the one hand, the study was conducted with only male subjects. Previous studies have shown that females have significantly lower variability than males.^[51,52]^ Hence, the synergistic effects of the lower limb on toe position may differ between men and women and may be modulated by the levels of female sex hormones. Thus, the conclusions of this study cannot be applied to female subjects. On the other hand, our study only focused on the effect of the swing leg on toe height, but the stance leg also influenced the height of the swing toe.^[53]^ Furthermore, it is necessary to investigate the impact of external factors like fatigue, footwear choices, and terrain variations on both kinematic synergy and the risk of falls. Recent studies have found that appropriate inner air insole pressures significantly reduces peak plantar pressure and increases plantar gradient angle.^[54,55]^ However, it remains unclear whether this affects lower limb joint synergy during walking and the risk of falls. Therefore, further research is needed to refine our understanding of the influence of these factors on human movement mechanisms.

## 5. Conclusions

In conclusion, our study provides evidence that kinematic synergy is related to vertical toe position during the forward movement of the foot in the swing phase of gait. We observed that different walking speeds resulted in varying modifications of the kinematic synergies that stabilized the vertical trajectory of the foot. Specifically, fast walking exhibited lower synergy than medium and slow walking speeds during the entire swing phase, with the mid-swing phase showing the most significant differences. These findings contribute to a better understanding of the role of kinematic synergy in gait stability and have implications for the development of interventions aimed at improving gait stability and reducing the risk of falls.

## Author contributions

**Conceptualization:** Xuan Liu, Ye Liu.

**Data curation:** Xuan Liu, Jitong Liang.

**Formal analysis:** Xuan Liu, Jitong Liang.

**Methodology:** Xuan Liu, Jitong Liang, Ye Liu.

**Software:** Xuan Liu, Jitong Liang.

**Writing – original draft:** Xuan Liu, Jitong Liang, Ye Liu.

**Writing – review & editing:** Xuan Liu, Jitong Liang, Ye Liu.

## Supplementary Material


