# Supplementary material for: Effects of walking speeds on lower extremity kinematic synergy in toe vertical position control: An experimental study
Source: Medicine (Baltimore). 2024 May 3;103(18):e38024. doi: 10.1097/MD.0000000000038024 (PMC11062729; doi:10.1097/MD.0000000000038024)
Supplement: Supplementary file 1 [file medi-103-e38024-s001.pdf]

北京体育大学运动科学实验伦理委员会

批　准　书

批准号：2021060H

项目名称：步行过程中下肢关节协调模式变化及步速对其的影响

项目负责人：柳璇　　　　**职称：**其他　　　　**联系电话：**18810856771

负责研究单位： 北京体育大学

合作研究单位：

研究起止时间：2021 年 04 月-2022 年 04 月

拟申报项目类别：

评审意见：

研究项目“步行过程中下肢关节协调模式变化及步速对其的影响”

经伦理委员会审查：

研究者的资格、经验符合试验要求；研究方案符合科学性和伦理原则的要求；获得知情同意的方法适当；受试者可能遭受的风险程度与研究预期的受益相比合适。

同意开展该项目的研究。

北京体育大学运动科学实验伦理委员会

主任委员签字：[illegible]

联系电话：15810435675

2021 年 04 月 21 日
